# Role of *Glycine max* in improving drought tolerance in *Zanthoxylum bungeanum*

**DOI:** 10.7717/peerj.9040

**Published:** 2020-05-05

**Authors:** Zilong Li, Akash Tariq, Kaiwen Pan, Corina Graciano, Feng Sun, Dagang Song, Olusanya Abiodun Olatunji

**Affiliations:** 1 CAS Key Laboratory of Mountain Ecological Restoration and Bioresource Utilization & Ecological Restoration Biodiversity Conservation Key Laboratory of Sichuan Province, Chengdu Institute of Biology, Chinese Academy of Sciences, Chengdu, Sichuan, China; 2 University of Chinese Academy of Sciences, Beijing, China; 3 School of Pharmacy, Guizhou University of Traditional Chinese Medicine, Guizhou, China; 4 State Key Laboratory of Desert and Oasis Ecology, Xinjiang Institute of Ecology and Geography, Chinese Academy of Sciences, Urumqi, China; 5 Cele National Station of Observation and Research for Desert-Grassland Ecosystems, Cele, Urumqi, China; 6 Key Laboratory of Biogeography and Bioresource in Arid Zone, Chinese Academy of Sciences, Urumqi, Xinjiang, China; 7 Xinjiang Desert Plant Roots Ecology and Vegetation Restoration Laboratory, Xinjiang Institute of Ecology and Geography, Chinese Academy of Sciences, Urumqi, China; 8 Instituto de Fisiología Vegetal, Consejo Nacional de Investigaciones Científicas y Técnicas Universidad Nacional de La Plata, Buenos Aires, Argentina; 9 Biogas Institute of Ministry of Agriculture and Rural Affairs, Chengdu, China; 10 Key Laboratory for Humid Subtropical Eco-Geographical Processes of the Ministry of Education, Fujian Normal University, Fuzhou, China

**Keywords:** *Zanthoxylum bungeanum*, Drought, Resistance, Intercropping, Soybean

## Abstract

Intercropping may improve community stability and yield under climate change. Here, we set up a field experiment to evaluate the advantages of cultivating *Z anthoxylum bungeanum* with *Capsicum annum*, and *Z. bungeanum* with *Glycine max* as intercrops, compared with cultivating *Z. bungeanum* in monoculture. Effects of extreme drought stress conditions on morphological, physiological, and biochemical traits of the three crop species cultivated in the three contrasting planting systems were compared. Results showed that extreme drought conditions induced negative impacts on *Z*.* bungeanum* grown in monoculture, due to reduced growth and metabolic impairment. However, limited stomatal conductance, reduced transpiration rate (*T_r_*), and increased water use efficiency, carotenoid content, catalase activity, and accumulation of soluble sugars in *Z*.* bungeanum* indicated its adaptive strategies for tolerance of extreme drought stress conditions. Compared with cultivation in monoculture, intercropping with *C. annum* had positive effects on *Z*.* bungeanum* under extreme drought stress conditions, as a result of improved crown diameter, leaf relative water content (LRWC), net photosynthetic rate, and proline content, while intercropping with *G. max* under extreme drought stress conditions increased net CO_2_ assimilation rates, LRWC, *T_r_*, and superoxide dismutase (SOD) activity. In conclusion, *Z*.* bungeanum* has an effective defense mechanism for extreme drought stress tolerance. Intercropping with *G. max* enhanced this tolerance potential primarily through its physio-biochemical adjustments, rather than as a result of nitrogen fixation by* G. max*.

## Introduction

With climate change, the magnitude and frequency of extreme drought events has significantly increased in most parts of the world (Beier et al., 2012; Dai, 2012; Liu et al., 2015; Smith, 2011). Drought profoundly impacts all components of the biosphere, from the individual to ecosystem scale (Garg, Burman & Kathju, 2004; He & Dijkstra, 2014; Jentsch & Beierkuhnlein, 2008; Ledger et al., 2012; Sicher, Timlin & Bailey, 2012), and affects economic income and human well-being (He & Dijkstra, 2014). Studies have shown the frequency of extreme drought events in most parts of China has increased and tends to be severe (Ayantobo, Li & Song, 2017; Jiang et al., 2017); therefore, it is important to understand and mitigate impacts on food production of these increasingly frequent severe drought conditions.

In the context of climate-mediated changes in precipitation patterns, some studies have focused on community and ecosystem stability (Isbell, Polley & Wilsey, 2009; Saxon, 2003) in response to predicted increases in extreme drought events that will likely alter ecosystem processes (Sun et al., 2019). In plants, for example, extreme drought affects transportation of metabolites and enzymatic reactions and hampers hydrolytic breakdown processes of proteins, lipids, and carbohydrates as during accumulation of reactive oxygen species (ROS) that leads to lipid peroxidation of cells. High levels of ROS not only cause damage to proteins, lipids, and carbohydrates, but also act as signal molecules (Veselin et al., 2015) to target genes that are subsequently up-regulated and increase plant resilience to drought stress (Mahajan & Tuteja, 2005; Puranik et al., 2011). Increased levels of anti-oxidation are associated with plant tolerance to external stressors (Pan et al., 2016), so it is important to identify effective antioxidant strategies in crop plants to improve tolerance to drought stress conditions.

Increasing the diversity of agricultural systems is an important strategy in sustainable agriculture to mitigate impacts of abiotic stressors (Jackson, Pascual & Hodgkin, 2007; Scherr & Mcneely, 2008). One approach is the use of intercropping, because it has been shown to elicit positive effects on yields through suppression of pests and pathogens, increased water use efficiency and nutrient utilization, and improved soil conditions (Iqbal, Cheema & An, 2007; Keating & Carberry, 1993; Li et al., 2001). Thus, improving the efficiency of intercropping is urgently required. China contains 22% of the global human population, but only <9% of the land area, so intercropping may represent a strategy to increase crop production efficiency in that country (Tong, 1994). The presence of key species has been shown to buffer the impacts of reducing species richness due to agricultural production (Hooper et al., 2005). For example, legume (Fabaceae) crops may represent such key species, because they are known to be important in agroecosystem productivity and stability (Spehn et al., 2002). Normally, leguminous crops fix nitrogen (N) through symbiotic azotobacteria (Mosier, 2002; Yingchao et al., 2019) and provide additional nutrients to nearby crops (Chu, Shen & Cao, 2004; Xin-Ping Chen et al., 2011), and as a consequence, leaf nitrogen concentration in neighboring crops is affected (Temperton et al., 2007). However, drought negatively affects symbiotic nitrogen fixation by leguminous crops, through reduced phloem flow that facilitates nitrogenase activity in nitrogenated nodules (Marino et al., 2007; Serraj, Sinclair & Purcell, 1999). Thus, the role of leguminous species in water and nutrient use efficiencies, and tolerance of neighboring tree crop species to extreme drought conditions is poorly understood, but urgently needed.

*Zanthoxylum bungeanum* (Rutaceae), which is distributed in tropical and subtropical regions (Feng et al., 2017), is a woody shrub that possesses medicinal properties against toothache and rheumatism, and is also used as a condiment (Yuan et al., 2017). The species tends to grow in arid environments, and the ongoing increases in extreme drought events have led to declines in yield and quality, and increases in mortality (Sun et al., 2016; Wang, 2016). Soybean (*Glycine max*) is a leguminous crop that benefits in growth from its ability to fix N symbiotically (Yang et al., 2018), and is commonly used in agroforestry systems, mainly to increase land use efficiency and crop yield (Echarte et al., 2011; Gong et al., 2014); it is one of the most well-studied crops under drought stress (Manavalan et al., 2009). In contrast, hot pepper (*Capsicum annum*: Solanaceae), which is sensitive to low levels of moisture (Qin et al., 2014), is an important horticultural crop widely cultivated in China, India, Indonesia, and Thailand, due to its enriched levels of antioxidants, high pungency, rich flavor, and vitamin content (Jeeatid et al., 2018; Sahitya et al., 2018). Here, we compared effects of intercropping *Z. bungeanum* with soybean or *C. annum* under extreme drought conditions to test the hypotheses that inter-cropping with *G. max* and *C. annum* will induce difference drought stress responses in *Z. bungeanum*, and that *G. max*, as a legume, will facilitate N uptake and enhance the tolerance of *Z. bungeanum* to extreme drought conditions.

## Materials and Methods

### Study site description

The study site, characterized by the Udic Luvisol soils, was located in Mao County (31°41′N, 103°53′E, 1,686 m a.s.l.), in southwestern China, where mean annual temperature is 8.9 °C, lowest mean annual temperature is –11.6 °C, and highest mean annual temperature is 32.3 °C; mean annual precipitation is 920 mm that mainly occurs during the period May–September (data collected at Mao County Ecological Station of Chinese Academy of Science). Extreme drought conditions in this region have increased significantly in recent years (Xia et al., 2018). Permission to conduct the study and collect samples at the field site was obtained from the farmer, Mr. Li Xingfu.

### Experimental design

The experiment was conducted from 1−30 August 2015 as a randomized block experiment, comprising three replicates of six treatments applied to 2.6 × 2.6-m plots. The experimental plots were covered with a rainproof shield constructed as a 2 m high steel frame with a transparent plastic roof (Borken et al., 2006); there was a 1-m wide buffer area between each plot and plots were surrounded by plastic partitions to prevent flow of surface and soil water.

Treatments comprised application of average rainfall (control: CK) or extreme drought conditions to (i) *Z. bungeanum* monoculture (Z); (ii) intercropping of *Z. bungeanum* and *G. max* (Z-G) and (iii) intercropping of *Z. bungeanum* and *C. annuum* (Z-C). *Z. bungeanum* seedlings with the same basal stem and plant height were selected as experimental materials and planted in the center of the plots in April 2013, and *G. max* and *C. annuum* were evenly planted at a density of 0.27 m^−2^ (25 plants) per plot in April 2015; plots were kept weed-free. Extreme drought is defined as continuous precipitation of <0.1 mm day^−1^ in August (*Zhiyuan* Wang et al., 2015); thus, the extreme drought treatment comprised no watering. The CK was the average rainfall in August (1983–2013) at the study site of 3.0 mm day^−1^ (Ng et al., 2015); CK plots were evenly irrigated twice a day (07:00–09:00 and 18:00–20:00, 1–30 August 2015) to allow for water loss through transpiration and evaporation.

On 30 August 2015, height (cm) of the experimental plants was recorded and crown size was measured as the average of two perpendicular transverse widths (Sánchez-González, ICRD & GM, 2007). Fully expanded leaves were sampled from the experimental plants, quickly placed in liquid N_2_, and returned to laboratory for storage at −80 ° C prior to analysis.

### Leaf relative water content

We measured leaf relative water content (LRWC) using the method described by Galle, Haldimann & Feller (2007) and calculated as LRWC = [(FW-DW)/(TW-DW)] ×100%, where FW is fresh weight of a single, fully expanded leaf per plant and DW is dry weight. After FW of the fully expanded leaves had been recorded, the samples were immediately dipped in distilled water, in the dark, at 4 °C. After 4 h, leaves were weighed to obtain turgor weight (TW), and then dried in an oven for 24 h at 70°; to determine DW.

### Pigment and photosynthetic parameters

Pigment was measured from 0.2 g of fresh leaves that were placed in five mL of 100% acetone for 36 h at room temperature in the dark. Then, reflectance of the extracting solution was determined at 662, 645, and 470 nm using a spectrophotometer and calculation of pigment content was based on the method reported by Liu et al. (2014).

Net CO_2_ assimilation rate (*P*_n_), stomatal conductance (*G*_s_), intercellular CO_2_concentration (*C*_i_), and transpiration rate (*T*_*r*_) were measured on fully expanded leaves at the end of the experiment between 09:00 and 11:00 hrs using a portable photosynthetic instrument (LI-6400, LI-COR Inc., USA). Photon flux density, CO_2_ concentration, and air relative humidity were maintained at 800 µmol m^−2^ s^−1^, 400 µmol mol^−1^, and 70–80% respectively. Intrinsic water use efficiency (WUE_intr_) was determined as WUE_intr_ = *P*_n_/*T*_*r*_, and *L*_*s*_ was calculated as *L*_*s*_ = 100% × (*C*_*a*_–*C*_*i*_)/*C*_*a*_.

### Soluble sugars, soluble protein, and proline

A supernatant was created from 0.2 g of DW leaves that were extracted three times using 6 ml of 80% ethanol at 80 °C for 30 min. Then, content of soluble sugars was separated with linear gradient from 50 to 150 Mm and determined following the method reported by Quan et al. (2004) and soluble protein content was determined using Bradford G-250 reagent. Proline was extracted from DW leaves using two mL of 10% acetic acid and five mL of 3% sulfosalicylic acid; proline content of the final supernatant was determined using the method described by Liu et al. (2014).

### ROS and membrane lipid peroxidation

Superoxide anion (O_2_^−^) production rate in leaves was determined from oxidation of hydroxylamine (Zhou et al., 2004) that was extracted from 0.2 g of DW leaves using two mL of 65 mM phosphate buffer (pH 7.8) followed by centrifugation at 5,000 g for 10 min. Then, 0.1 mL of supernatant was incubated at 25 °C for 20 min in 0.9 mL of 65 Mm phosphate buffer (pH 7.8), 0.1 mL of 10 mM hydroxylammonium chloride; next, 17 mM sulphanilamide and 7 mM *α*-naphthylamine were added to the mixture and incubated again at 25 °C for 20 min. Ethyl ether at the same volume was added to the mixture that was then centrifuged at 1,500 g for 5 min, and absorbance was read at 530 nm using a spectrophotometer (UV 330, Perkin Elmer, USA).

Content of hydrogen peroxide (H_2_O_2_) was determined by measuring absorbance of the titanium–peroxide complex at 410 nm (Zhou et al., 2004). Fresh leaves (0.2 g) were ground with five mL of acetone and centrifuged at 3,000 g for 10 min; then, 0.1 mL of titanium reagent (50 µL of 20% titanium tetrachloride in concentrated HCl), 0.2 mL of ammonia, and one mL of supernatant were added and the mixture was centrifuged at 3,000 g for 10 min. The resulting precipitate was washed five times with acetone and centrifuged at 10,000 g for 5 min, before it was dissolved in three mL of 1 M H_2_SO_4_; finally absorbance was measured at 410 nm using a spectrophotometer.

We used the thiobarbituric acid (TBA) method (Zhou, Lam & Zhang, 2007) to determine malondialdehyde (MDA) content. We ground 0.2 g of fresh leaves in two mL of 50 mM phosphate buffer (pH 7.8) that was then centrifuged at 12,000 g for 20 min. Next, one mL of the supernatant was mixed with three mL of 20% trichloroacetic acid solution that contained 2% TBA, and the reaction mixture was incubated in water at 95 °C for 30 min before it was cooled quickly in an ice bath and centrifuged at 15,000 g for 10 min. Absorption at 532 and 600 nm was determined using a spectrophotometer, and MDA content was calculated according to an extinction coefficient of 155 mM^−1^ cm^−1^.

### Antioxidant enzyme activity

The NBT method (Fu & Huang, 2001) was used to determine superoxide dismutase (SOD) activity, where one unit of SOD activity was defined as the amount of enzyme required for 50% inhibition of NBT reduction at 560 nm. Activity of catalase (CAT) was measured using the method described by Fu & Huang (2001), by determining H_2_O_2_ decomposition per 1 min at 240 nm absorbance, where one unit of CAT activity was defined as change in absorbance of 0.01 units per min.

### Statistical analyses

We used two-way ANOVA to test for effects of cropping system and extreme drought on physio-biochemical parameters, and one-way ANOVA to test effects of all treatments on physio-biochemical parameters. Data were checked for normality and homogeneity of variances prior to ANOVA, and means were compared using Duncan’s test. Statistical analyses were conducted in SPSS v.17.0 (SPSS Inc., Chicago, IL). Presented data are means ± SE.

## Results

### Morphological traits

Overall, there was a strong reduction in the morphological traits in extreme drought stressed plants compared with the control across the three cropping systems (Table 1). Extreme drought reduced the crown diameter of *Z. bungeanum* across the three cropping systems (decreases of 60.27, 32.12, and 44.33% in Z, Z-C, and Z-G, respectively), but there was no effect on tree height. Although height and crown diameter of *C. annuum* and *G. max* decreased slightly after 30 d of extreme drought stress, there were no significant effects of drought (Table 2).

### Leaf relative water content, photosynthetic pigments, and gas exchange

*Z. bungeanum* LRWC was slightly reduced by extreme drought across the three cropping systems. We found that the LRWC content in *Z. bungeanum* was significantly lower in the cropping system, regardless of drought conditions, than in the other two systems (Table 1) and LRWC in *C. annuum* was greater than in *G. max* (Table 2). There was no effect of extreme drought stress on chlorophyll b (Chl b) or carotenoid (Car) content, but it decreased Chl a content in *Z. bungeanum* (Table 1). Irrespective of extreme drought, content of Chl a, Chl b, and Car in *G. max* was greater than in *C. annuum* (Table 2). Extreme drought led to a decrease in Car in *G. max*, but there were not effects on Chl a or Chl b (Table 1). In general, extreme drought decreased photosynthesis and increased *L*_*s*_ in *Z. bungeanum* across the three cropping systems. There were no effects of extreme drought on *C*_*i*_or *G*_*s*_ in *Z. bungeanum* across the three cropping systems; however, there were decreases in *T*_*r*_ in *Z. bungeanum* in Z and Z-C. WUE_intr_ of *Z. bungeanum* in Z was greater under extreme drought conditions. There were no effects of extreme drought on photosynthetic parameters in *C. annuum* or *G. max* across the three cropping systems (Table 2).

### Antioxidant defense system

**Figure 1:**
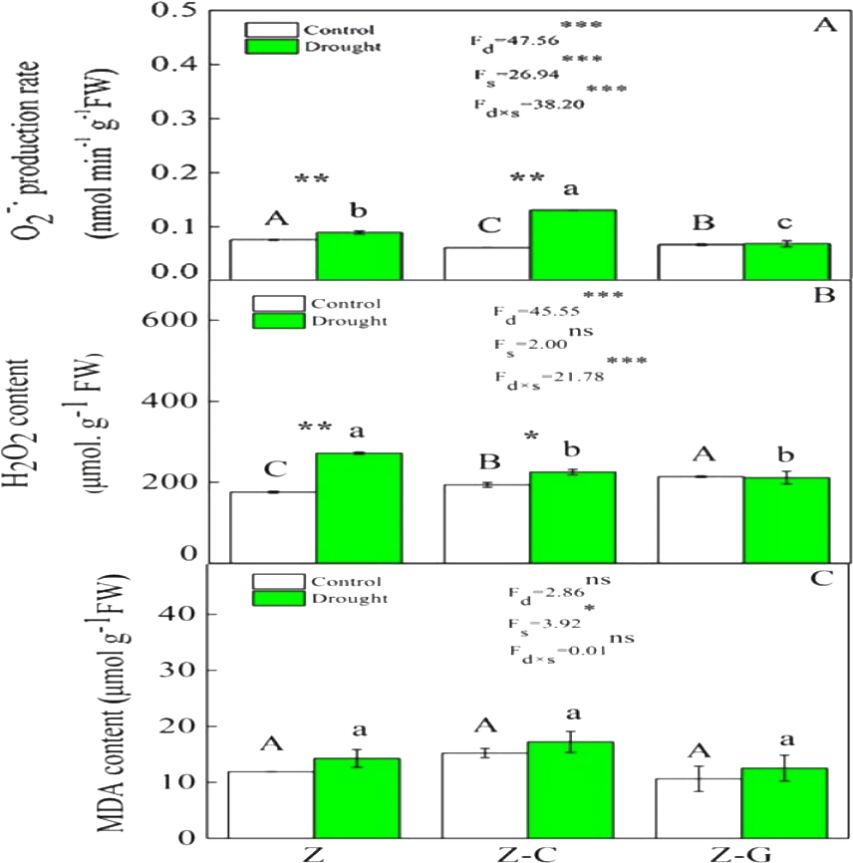
Effects on the ROS content of *Z. bungeanum* under extreme drought. “Z-G” denotes *Z. bungeanum* intercropping with *G. max*, “Z-C” denotes *Z. bungeanum* intercropping with *C. annuum*, and “Z” denotes the *Z*. *bungeanum* monoculture. Vertical bars show  ± SE of the mean (*n* = 3). Different uppercase letters indicate significant differences among control (normal rainfall) treatments. Different lowercase letters indicate significant differences among extreme drought treatments. “d” denotes extreme drought; “s” denotes planting system; “d ×s” denotes the interaction of extreme drought and planting system; Proportion of explained variance by extreme drought and planting system effects and by their interactions (two-way ANOVA). Significant levels:^∗^*P* < 0.05,^∗∗^*P* < 0.01,^∗∗∗^*P* < 0.001, “ns” no significant.

**Figure 2:**
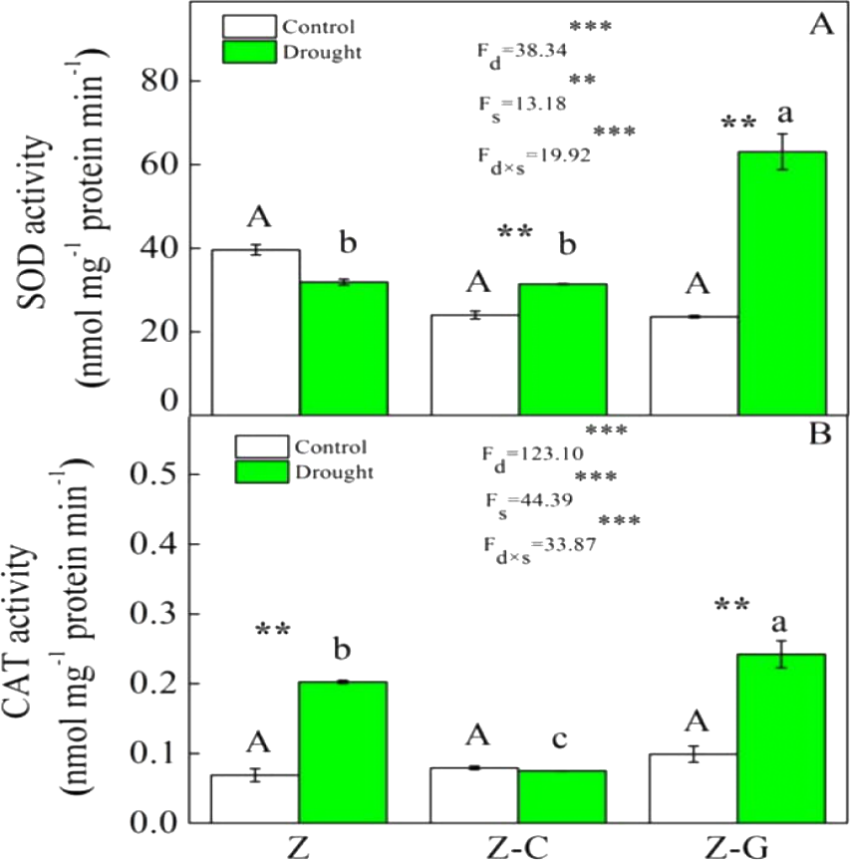
Effects on the SOD and CAT activity of *Z. bungeanum* under extreme drought. “Z-G” denotes *Z. bungeanum* intercropping with *G. max*, “Z-C” denotes *Z. bungeanum* intercropping with *C. annuum*, and “Z” denotes the *Z*. *bungeanum* monoculture. Vertical bars show  ± SE of the mean (*n* = 3) Different uppercase letters indicate significant differences among control (normal rainfall) treatments. Different lowercase letters indicate significant differences among extreme drought treatments. “d” denotes extreme drought; “s” denotes planting system; “d ×s” denotes the interaction of extreme drought and planting system; Proportion of explained variance by extreme drought and planting system effects and by their interactions (two-way ANOVA). Significant levels:^∗^*P* < 0.05,^∗∗^*P* < 0.01,^∗∗∗^*P* < 0.001, “ns” no significant.

Extreme drought increased the O^2−^ production rate and H_2_O_2_ content in *Z. bungeanum* in the Z and Z-C treatments, but there was no effect in the Z-G treatment. There was no effect of extreme drought stress on MDA content in *Z. bungeanum* in the three cropping systems (Fig. 1); however, extreme drought stress led to increased SOD activity in *Z. bungeanum* in Z-C and Z-G and greater CAT activity in Z and Z-G (Fig. 2). Content of H_2_O_2_ in *C. annuum* and MDA in *G. max* were greater under extreme drought conditions, and there was no effect on SOD or CAT activity (Table 2).

**Table 1:** Growth, photosynthetic parameters and osmotic adjustment matter content of of *Z. bungeanum* under extreme drought.

**Plant system**	**Z**		**Z-C**		**Z-G**				
**Parameters**	**Control**	**Drought**	**Control**	**Drought**	**Control**	**Drought**	**System**	**Drought**	System^*^ Drought
Height (cm)	161.50 ± 3.75A	158.50 ± 5.48A	180.00 ± 15.31A	149.00 ± 28.29A	158.00 ± 27.51A	136.00 ± 24.76A	**ns**	**ns**	**ns**
Crown diameter (cm)	100.67 ± 5.36A	40.00 ± 3.46C	100.67 ± 9.82A	68.33 ± 15.25C	110.78 ± 1.15A	61.67 ± 15.34B	**ns**	^***^	**ns**
Chl a (mg/g)	4.86 ± 0.47A	3.92 ± 0.00A	4.69 ± 0.10A	4.21 ± 0.31A	4.97 ± 0.36A	4.37 ± 0.18A	**ns**	^*^	**ns**
Chl b (mg/g)	2.25 ± 0.06A	2.19 ± 0.29A	2.86 ± 0.22A	2.86 ± 0.10A	1.70 ± 0.30A	1.78 ± 0.13A	^**^	**ns**	**ns**
Car (mg/g)	1.73 ± 0.12A	1.92 ± 0.14A	1.91 ± 0.22A	2.03 ± 0.14A	1.90 ± 0.04A	1.90 ± 0.04A	**ns**	**ns**	**ns**
LRWC (%)	86.15 ± 2.08A	74.74 ± 1.61AB	66.59 ± 5.85BC	62.24 ± 5.74C	85.65 ± 1.78A	84.50 ± 2.04A	^**^	**ns**	^*^
*P*_*n*_ (µmol m^−2^s^−1^)	15.36 ± 0.03AB	12.75 ± 0.83C	16.39 ± 0.01A	13.71 ± 1.08BC	13.93 ± 0.87BC	11.77 ± 0.81C	^*^	^**^	**ns**
*C*_*i*_(µmol mol^−1^)	305.06 ± 5.47A	291.73 ± 10.92A	301.20 ± 17.65A	295.94 ± 5.76A	302.40 ± 10.86A	289.45 ± 3.88A	**ns**	**ns**	**ns**
*G*_*s*_ (mol m^−2^s^−1^)	0.34 ± 0.02AB	0.31 ± 0.04AB	0.31 ± 0.01AB	0.29 ± 0.01B	0.37 ± 0.03A	0.35 ± 0.00AB	^*^	**ns**	**ns**
*T*_*r*_ (mmol m^−2^ s^−1^)	3.55 ± 0.18AB	2.35 ± 0.16D	3.70 ± 0.03A	2.79 ± 0.10C	3.12 ± 0.07BC	2.86 ± 0.21C	**ns**	^***^	**ns**
WUE_intr_ (µmol mol^−1^)	4.35 ± 0.23B	5.41 ± 0.03A	4.43 ± 0.04AB	4.96 ± 0.57AB	4.47 ± 0.33AB	4.14 ± 0.27B	**ns**	**ns**	**ns**
*L*_*s*_	0.77 ± 0.01B	0.86 ± 0.02A	0.73 ± 0.01B	0.85 ± 0.03A	0.78 ± 0.01B	0.89 ± 0.03A	**ns**	^***^	**ns**
Soluble sugar (mg g^−1^ DW)	0.56 ± 0.02B	0.86 ± 0.13AB	0.90 ± 0.13AB	0.90 ± 0.10AB	1.05 ± 0.00AB	1.35 ± 0.31A	^*^	**ns**	**ns**
Soluble protein (mg g^−1^ DW)	23.43 ± 1.34B	21.40 ± 0.20B	30.80 ± 0.78A	24.75 ± 0.35B	24.70 ± 2.51B	11.25 ± 0.46C	^***^	^***^	^**^
Proline (µg g^−1^ DW)	0.28 ± 0.01A	0.26 ± 0.01AB	0.26 ± 0.00AB	0.27 ± 0.00A	0.25 ± 1.15B	0.26 ± 0.01AB	**ns**	**ns**	^*^

**Notes.**

“Z-G” denotes *Z. bungeanum* intercropping with *G. max*, “Z-C” denotes *Z. bungeanum* intercropping with *C. annuum*, and “Z” denotes the *Z*. *bungeanum* monoculture. Different uppercase letters indicate significant differences among all treatments. Two-way ANOVA was used to test the effects of planting systems and extreme drought on the growth and pigment contents.

* *P* < 0.05.

** *P* < 0.01.

*** *P* < 0.001.

ns non-significant (*P* > 0.05).

**Table 2:** Growth, photosynthetic parameters, the content of free radical, enzyme activity and osmotic adjustment matter content of *C. annuum and G.max* under extreme drought.

**Plant system**	***C. annuum***		***G. max***	
**Parameters**	**Control**	**Drought**	**Control**	**Drought**
Height (cm)	25.67 ± 0.35A	25.00 ± 1.20A	29.93 ± 0.98A	27.80 ± 3.12A
Crown diameter (cm)	27.44 ± 1.06A	19.22 ± 3.29A	25.44 ± 2.26A	24.33 ± 3.36A
Chl a (mg/g)	4.32 ± 0.39B	4.64 ± 0.03B	6.59 ± 0.48A	5.82 ± 0.19A
Chl b (mg/g)	2.23 ± 0.23A	2.36 ± 0.10A	2.67 ± 0.18A	2.54 ± 0.14A
Car (mg/g)	1.64 ± 0.25B	2.04 ± 0.12B	2.77 ± 0.06A	1.98 ± 0.06B
LRWC (%)	89.41 ± 0.03A	81.86 ± 0.05A	49.05 ± 9.84B	55.38 ± 5.09B
*P*_*n*_ (µmol m^−2^s^−1^)	16.04 ± 0.68A	16.68 ± 1.21A	14.26 ± 1.48A	12.63 ± 1.43A
*C*_*i*_(µmol mol^−1^)	307.56 ± 16.04A	297.69 ± 11.36A	309.62 ± 10.38A	306.20 ± 7.25A
*G*_*s*_ (mol m^−2^s^−1^)	0.37 ± 0.05A	0.51 ± 0.09A	0.48 ± 0.01A	0.37 ± 0.01A
*T*_*r*_ (mmol m^−2^ s^−1^)	2.99 ± 0.94A	4.03 ± 0.62A	3.99 ± 0.51A	3.71 ± 0.43A
WUE_intr_ (µmol mol^−1^)	4.30 ± 0.49A	3.80 ± 0.34A	4.34 ± 0.43A	3.18 ± 0.51A
H_2_O_2_content (µmol g^−1^ FW)	126.36 ± 16.89B	265.28 ± 40.96A	53.94 ± 17.20B	77.40 ± 5.31B
O_2_^−^ producing rate (nmol min^−1^ g^−1^ FW)	0.03 ± 0.005A	0.03 ± 0.025A	0.052 ± 0.019A	0.039 ± 0.009A
MDA content (µmol g^−1^ FW)	8.50 ± 1.11B	12.79 ± 0.32B	12.28 ± 1.38B	20.32 ± 2.23A
SOD activity (nmol mg^−1^ protein min^−1^)	22.79 ± 1.43A	23.16 ± 0.77A	30.21 ± 5.49A	26.41 ± 3.53A
CAT activity (nmol mg^−1^ protein min^−1^)	0.008 ± 0.003B	0.024 ± 0.003B	0.085 ± 0.007A	0.095 ± 0.027A
Soluble sugar (mg g^−1^ DW)	0.63 ± 0.05A	0.56 ± 0.11A	1.18 ± 0.37A	0.79 ± 0.06A
Soluble protein (mg g^−1^ DW)	31.57 ± 1.16A	31.23 ± 0.33A	26.32 ± 4.98A	26.03 ± 3.54A
Proline (µg g^−1^ DW)	0.04 ± 0.004A	0.04 ± 0.002A	0.04 ± 0.005A	0.03 ± 0.003A

**Notes.**

“Z-G” denotes *Z. bungeanum* intercropping with *G. max*, “Z-C” denotes *Z. bungeanum* intercropping with *C. annuum*, and “Z” denotes the *Z*. *bungeanum* monoculture. Different uppercase letters indicate significant differences among all treatments. ANOVA was used to assess the effects of extreme drought on plant physiology properties.

* *P* < 0.05.

** *P* < 0.01.

*** *P* < 0.001

ns non-significant (*P* >0.05).

### Osmolytes and soluble proteins

Content of soluble sugar and proline in *Z. bungeanum* did not vary with cropping system under extreme drought stress conditions; however, content of soluble protein of *Z. bungeanum* was reduced in Z-C and Z-G (Table 1). There were no effects of extreme drought conditions on soluble sugar, soluble protein or proline content in *C. annuum* and *G. max* (Table 2).

## Discussion

### Plant growth, LRWC, pigment content, and gas exchange

Drought is the principal abiotic stress factor that limits agricultural crop productivity, growth, and development by lowering plant nutrient uptake from arid soils due to reduced expansion of plant cells (Ihsan et al., 2016; Luo et al., 2010). As shown in Tables 1 and 2, short-term extreme drought had no effect on height of *Z. bungeanum*, *C. annuum*, or *G. max*, indicating that plant height is a long-term nutrient accumulation process (Table 2). However, we found that extreme drought reduced *Z. bungeanum* crown diameter in Z, Z-C and Z-G cropping systems (Table 1). Water deficit reduces soil nutrient availability and plant nutrient uptake, and has a negative effects on soil microbe activities (Hu & Schmidhalter, 2001). Our experiment showed that crown diameter in *Z. bungeanum* is sensitive to extreme drought conditions that will then likely affect levels of light radiation interception.

Leguminous crops may provide additional nutrients to nearby crops, and our previous study (Sun et al., 2019) showed that the Z-G treatment led to lower content of soil and leaf N content than the Z-C and Z treatment. This effect is probably due to the sensitivity of synergetic N fixation to drought during the reproductive stage that leads to reductions in N accumulation in leguminous crops (Serraj, Sinclair & Purcell, 1999), and the greater sensitivity of N_2_ fixation to drought stress than N uptake and assimilation in soil (Purcell & Andyking, 1996).

Plant pigment is a receptor of light radiation, and its level of content directly affects intensity of photosynthesis and formation of dry matter (Ghosh et al., 2004). Declines in Chl content commonly occur under drought stress (Bijanzadeh & Emam, 2010; Din et al., 2011), indicating effects on the pigment synthesis pathway or pigment degradation (Batra, Sharma & Kumari, 2014). However, we found there were no effects of extreme drought conditions on pigment content in *Z. bungeanum* in any of the three cropping systems, indicating that short-term extreme drought conditions did not affect the synthesis of chlorophyll encoded by the Cab gene family (Din et al., 2011). Efficient N nutrition alleviates effects of extreme drought conditions, by maintaining normal crop metabolism, even under low tissue water condition (Abid et al., 2016), and we found that Car content in *G. max* decreased under extreme drought conditions (Table 2), possibly due to reductions in NH_4_^+^-N and NO_3_^−^-N absorption.

Leaf growth reflects the physiological state of the plant when there is a water deficit (Parida et al., 2007), and LRWC directly reflects leaf water status. We found that LRWC of *Z. bungeanum* was lower in the Z monoculture than in the two intercropping systems (Table 1); however, the slight decreases in *Z. bungeanum* LRWC in Z-C and Z-G indicate that increasing diversity of crops may increase the water-holding capacity of the main crop species. Water-holding capacity directly affects plant metabolism, and leaf water status is closely related to photosynthetic activity (Ramachandra, Chaitanya & Vivekanandan, 2004). Our findings revealed that *P*_*n*_ in *Z. bungeanum* decreased in the Z monoculture and Z-C intercropping system under extreme drought stress conditions (Table 2), indicating that intercropping with *G. max* not only enhanced the water holding capacity of *Z. bungeanum*, but also promoted its photosynthetic capacity under extreme drought stress conditions. A key driver of decline in photosynthetic capacity is stomatal closure (Cornic, 2000); however, it is unclear whether drought inhibits photosynthesis due to stomatal closure or other metabolic impairments (Tezara et al., 1999). We found that extreme drought stress conditions led to increases in *L*_*s*_ in the three cropping systems and reduced levels of *C*_*i*_ in all three species, indicated that the decrease in photosynthetic capacity was related to water holding capacity and stomatal closure. Intercropping with *G. max* facilitated the maintenance of levels of *P*_*n*_ and *G*_*s*_ in *Z. bungeanum* under extreme drought conditions, indicating that stable LRWC allows normal photosynthetic activity to continue. Intercrops tend to have high water-use efficiency compared with single cropping (Morris & Garrity, 1993); however, our experiment showed there were high levels of WUE_intr_ in *Z. bungeanum* in the Z monoculture _,_probably in response to drought. The lack of effect of extreme drought on levels of *P*_*n*_ in *Z. bungeanum* in the Z-G mixed culture showed that intercropping with *G. max* may alleviate declines in *Z. bungeanum* photosynthetic activity under extreme drought stress conditions. We also found that *G*_*s*_ in *G. max* decreased under extreme drought conditions, indicating that water absorption was blocked that may have reduced competition for soil water between *G. max* and *Z. bungeanum*. Supporting our findings, previous work has shown that levels of *T*_*r*_ in *G. max* decrease under drought stress (He et al., 2017). Extreme drought conditions led to decreases in *P*_*n*_ in *Z. bungeanum*, but no changes in WUE_intr_ or *G*_*s*_ in the Z-C intercropping system. However, we found that compared with Z-G, levels of *T*_*r*_ in *Z. bungeanum* were greater in the Z-C intercropping system, indicating greater water demand in *C. annuum* and competition for water between *C. annuum* and *Z. bungeanum* that likely affected levels of water absorption in *Z. bungeanum*.

### ROS production and antioxidant enzymes

Plants that suffer drought stress accumulate free radicals, including superoxide anion, and hydrogen peroxide (Munné-Bosch & Penuelas, 2003), and sudden rises in ROS production induce DNA breakage, lipid peroxidation, protein degradation, and ultimately, cell death (Apel & Hirt, 2004). Responses of *Z. bungeanum* to drought stress conditions include increases in MDA content (Duling & Liu, 2010), and in this experiment, we found that *Z. bungeanum* ROS (O^2−^ and H_2_O_2_) production and MDA content were greater under extreme drought stress conditions in the Z-C intercropping system than in the control (Fig. 1). Similarly, SOD activity was greater in the Z-C intercropping system, supporting previous findings that elevated levels of SOD activity improve tolerance of oxidative stress (Anikó Mátai & Éva, 2019; Oliveira et al., 2014; Pan, Wu & Yu, 2006) and demonstrating antioxidant defense mechanisms are active in *Z. bungeanum* under drought conditions. We found that H_2_O_2_ and MDA content were also greater in *C. annuum* under extreme drought conditions. In the Z-G intercropping systems, there were no effects of extreme drought on ROS or MDA content in *Z. bungeanum*; however, SOD activity in *Z. bungeanum* (Fig. 2) and MDA content in *G. max* were greater, indicating that *G. max* facilitates greater drought tolerance in *Z. bungeanum* than when *Z. bungeanum* is grown in a monoculture or with *C. annuum*.

### Soluble sugars, proline and soluble proteins

Under normal moisture conditions, content of soluble sugars and soluble protein in *Z. bungeanum* were found to be greater in the Z-C intercropping system than in the Z monoculture or Z-G intercropping system, while proline content was greater in the Z monoculture than in the Z-G intercropping system. In contrast, under extreme drought conditions, soluble sugars and soluble protein content in *Z. bungeanum* was greater in the Z monoculture and Z-C intercropping system than in the Z-G treatment. We found that extreme drought conditions had no effect on content of soluble sugars, soluble protein, or proline in *C. annuum* and *G. max*.

When osmotic regulation reduces osmotic potential, plants accumulate a variety of osmolytes that allow the absorption of water by cells that is essential during prolonged drought for the maintenance of physiological activity (Farooq et al., 2009; Ramanjulu & Bartels, 2002). Short periods of drought stress increase soluble sugar content in leaves (Haiyan Wang et al., 2015), and our results showed that, under normal moisture conditions, soluble sugar and soluble protein content in *Z. bungeanum* was greatest in the Z-G intercropping system than the Z monoculture or Z-C treatment, but under extreme drought conditions, they were lower in the Z-G treatment. These results may reflect the drought-mediated limitation of normal utilization and transport of osmotic substances or subsequent hydrolysis of starch (Shubhra Dayal, Goswami & Munjal, 2004). Our results show that osmoregulation in *Z. bungeanum* under extreme drought conditions was lowest in the Z-C intercropping system.

## Conclusions

*Z. bungeanum* exhibits strong drought resistance, because of its high photosynthetic parameters, water use efficiency, and osmotic flexibility. We found that *G. max and C. annum* coped with extreme drought stress conditions by improving water holding capacity in *Z. bungeanum*, but by contrasting modes of action proving the first hypothesis of this study. Greater drought resistance in *Z. bungeanum* was facilitated by *G. max*, due to its positive effect on drought responses in *Z. bungeanum*, rather than through N fixation, which disapproves the second hypothesis of this study. There was evidence of water competition between *C. annum* and *Z. bungeanum*; therefore, we conclude the Z-G intercropping system facilitated better tolerance to extreme drought conditions for *Z. bungeanum* than the Z-C treatment.

##  Supplemental Information

10.7717/peerj.9040/supp-1 Supplemental Information 1 Raw data

## Additional Information and Declarations

### Field Study Permissions

The following information was supplied relating to field study approvals (i.e., approving body and any reference numbers): Permission to conduct the study and collect samples at the field site was obtained from the farmer, Mr. Li Xingfu.

### Data Availability

The following information was supplied regarding data availability: The raw measurements are available as a Supplementary File.

## References

[ref-1] Abid M, Tian Z, Ata-Ul-Karim ST, Cui Y, Liu Y, Zahoor R, Jiang D, Dai T (2016). Nitrogen nutrition improves the potential of Wheat (*Triticum aestivum* L.) to alleviate the effects of drought stress during vegetative growth periods. Frontiers in Plant Science.

[ref-2] Anikó Mátai DN, Éva  H (2019). UV-B strengthens antioxidant responses to drought in *Nicotiana benthamiana* leaves not only as supplementary irradiation but also as pre-treatment. Plant Physiology and Biochemistry.

[ref-3] Apel K, Hirt H (2004). Reactive oxygen species: metabolism, oxidative stress, and signal transduction. Annual Review of Plant Biology.

[ref-4] Ayantobo OO, Li Y, Song S (2017). Spatial comparability of drought characteristics and related return periods in mainland China over 1961–2013. Journal of Hydrology.

[ref-5] Batra NG, Sharma V, Kumari N (2014). Drought-induced changes in chlorophyll fluorescence, photosynthetic pigments, and thylakoid membrane proteins of Vigna radiata. Journal of Plant Interactions.

[ref-6] Beier C, Beierkuhnlein C, Wohlgemuth T, Penuelas J, Emmett B, Korner C, De Boeck H, Christensen JH, Leuzinger S, Janssens IA, Hansen K (2012). Precipitation manipulation experiments—challenges and recommendations for the future. Ecology Letters.

[ref-7] Bijanzadeh E, Emam Y (2010). Effect of defoliation and drought stress on yield components and chlorophyll content of wheat. Pakistan Journal of Biological Sciences.

[ref-8] Borken W, Savage K, Davidson EA, Trumbore S (2006). Effects of experimental drought on soil respiration and radiocarbon efflux from a temperate forest soil. Global Change Biology.

[ref-9] Chu GX, Shen QR, Cao KL (2004). Nitrogen fixation and N transfer from peanut to rice cultivated in aerobic soil in an intercropping system and its effect on soil N fertility. Plant Soil.

[ref-10] Cornic G (2000). Drought stress inhibits photosynthesis by decreasing stomatal aperture-not by affecting ATP synthesis. Trends in Plant Science.

[ref-11] Dai A (2012). Increasing drought under global warming in observations and models. Nature Climate Change.

[ref-12] Din J, Khan SU, Ali I, Gurmani AR (2011). Physiological and agronomic response of canola varieties to drought stress. The Journal of Animal & Plant Sciences.

[ref-13] Duling L, Liu S (2010). Comparative drought resistance of different varieties of *Zanthoxylum bungeanum*. Agricultural Research in the Arid Areas.

[ref-14] Echarte L, Maggiora AD, Cerrudo D, Gonzalez VH, Abbate P, Cerrudo A, Sadras VO, Calviño P (2011). Yield response to plant density of maize and sunflower intercropped with soybean. Field Crops Research.

[ref-15] Farooq M, Wahid A, Kobayashi N, Fujita D, Basra SMA (2009). Plant drought stress: effects, mechanisms and management. Agronomy for Sustainable Development.

[ref-16] Feng S, Zhao L, Liu Z, Liu Y, Yang T, Wei A (2017). De novo transcriptome assembly of *Zanthoxylum bungeanum* using Illumina sequencing for evolutionary analysis and simple sequence repeat marker development. Scientific Reports.

[ref-17] Fu J, Huang B (2001). Involvement of antioxidants and lipid peroxidation in the adaptation of two cool-season grasses to localized drought stress. Environmental and Experimental Botany.

[ref-18] Galle A, Haldimann P, Feller U (2007). Photosynthetic performance and water relations in young pubescent oak (*Quercus pubescens*) trees during drought stress and recovery. New Phytologist.

[ref-19] Garg BK, Burman U, Kathju S (2004). The influence of phosphorus nutrition on the physiological response of moth bean genotypes to drought. Journal of Plant Nutrition and Soil Science.

[ref-20] Ghosh PK, Ajay, Bandyopadhyay KK, Manna MC, Mandal KG, Misra AK, Hati KM (2004). Comparative effectiveness of cattle manure, poultry manure, phosphocompost and fertilizer-NPK on three cropping systems in vertisols of semi-arid tropics. II. Dry matter yield, nodulation, chlorophyll content and enzyme activity. Bioresource Technology.

[ref-21] Gong W, Qi P, Du J, Sun X, Wu X, Song C, Liu W, Wu Y, Yu X, Yong T, Wang X, Yang F, Yan Y, Yang W (2014). Transcriptome analysis of shade-induced inhibition on leaf size in relay intercropped soybean. PLOS ONE.

[ref-22] He J, Du YL, Wang T, Turner NC, Yang RP, Jin Y, Xi Y, Zhang C, Cui T, Fang XW, Li FM (2017). Conserved water use improves the yield performance of soybean (Glycine max (L.) Merr.) under drought. Agricultural Water Management.

[ref-23] He M, Dijkstra FA (2014). Drought effect on plant nitrogen and phosphorus: a meta-analysis. New Phytologist.

[ref-24] Hooper DU, Chapin FS, Ewel JJ, Hector A, Inchausti P, Lavorel S, Lawton JH, Lodge DM, Loreau M, Naeem S, Schmid B, Setälä H, Symstad AJ, Vandermeer J, Wardle DA (2005). Effects of biodiversity on eosystem functioning: a consensus of current knowledge. Ecological Monographs.

[ref-25] Hu Y, Schmidhalter U (2001). Effects of salinity and macronutrient levels on micronutrients in wheat. Journal of Plant Nutrition.

[ref-26] Ihsan MZ, El-Nakhlawy FS, Ismail SM, Fahad S, Daur I (2016). Wheat phenological development and growth studies as affected by drought and late season high temperature stress under arid environment. Frontiers in Plant Science.

[ref-27] Iqbal J, Cheema ZA, An M (2007). Intercropping of field crops in cotton for the management of purple nutsedge (*Cyperus rotundus* L.). Plant Soil.

[ref-28] Isbell FI, Polley HW, Wilsey BJ (2009). Biodiversity, productivity and the temporal stability of productivity: patterns and processes. Ecology Letters.

[ref-29] Jackson LE, Pascual U, Hodgkin T (2007). Utilizing and conserving agrobiodiversity in agricultural landscapes. Agriculture, Ecosystems & Environment.

[ref-30] Jeeatid N, Techawongstien S, Suriharn B, Chanthai S, Bosland PW, Techawongstien S (2018). Influence of water stresses on capsaicinoid production in hot pepper (*Capsicum chinense* Jacq.) cultivars with different pungency levels. Food Chemistry.

[ref-31] Jentsch A, Beierkuhnlein C (2008). Research frontiers in climate change: effects of extreme meteorological events on ecosystems. Comptes Rendus Geoscience.

[ref-32] Jiang X, Shu J, Wang X, Huang X, Wu Q (2017). The roles of convection over the western maritime continent and the Philippine Sea in interannual variability of summer rainfall over Southwest China. Journal of Hydrometeorology.

[ref-33] Keating BA, Carberry PS (1993). Resource capture and use in intercropping: solar radiation. Field Crops Research.

[ref-34] Ledger ME, Brown LE, Edwards FK, Milner AM, Woodward G (2012). Drought alters the structure and functioning of complex food webs. Nature Climate Change.

[ref-35] Li L, Sun J, Zhang F, Li X, Yang S, Rengel Z (2001). Wheat/maize or wheat/soybean strip intercropping I. Yield advantage and interspecific interactions on nutrients. Field Crops Research.

[ref-36] Liu C, Wang Y, Pan K, Zhu T, Li W, Zhang L (2014). Carbon and nitrogen metabolism in leaves and roots of Dwarf Bamboo (*Fargesia denudata Yi*) subjected to drought for two consecutive years during sprouting period. Journal of Plant Growth Regulation.

[ref-37] Liu D, Ogaya R, Barbeta A, Yang X, Penuelas J (2015). Contrasting impacts of continuous moderate drought and episodic severe droughts on the aboveground-biomass increment and litterfall of three coexisting Mediterranean woody species. Global Change Biology.

[ref-38] Luo Y, Zhao X, Zhou R, Zuo X, Zhang J, Li Y (2010). Physiological acclimation of two psammophytes to repeated soil drought and rewatering. Acta Physiologiae Plantarum.

[ref-39] Mahajan S, Tuteja N (2005). Cold, salinity and drought stresses: an overview. Archives of Biochemistry & Biophysics.

[ref-40] Manavalan LP, Guttikonda SK, Tran LS, Nguyen HT (2009). Physiological and molecular approaches to improve drought resistance in soybean. Plant and Cell Physiology.

[ref-41] Marino D, Frendo P, Ladrera R, Zabalza A, Puppo A, Arrese-Igor C, Gonzalez EM (2007). Nitrogen fixation control under drought stress. Localized or systemic?. Plant Physiology.

[ref-42] Morris RA, Garrity DP (1993). Resource capture and utilization in intercropping: water. Field Crops Research.

[ref-43] Mosier AR (2002). Environmental challenges associated with needed increases in global nitrogen fixation. Nutrient Cycling in Agroecosystems.

[ref-44] Munné-Bosch S, Penuelas J (2003). Photo- and antioxidative protection, and a role for salicylic acid during drought and recovery in field-grown *Phillyrea angustifolia* plants. PLANTA.

[ref-45] Ng EL, Patti AF, Rose MT, Schefe CR, Smernik RJ, Cavagnaro TR (2015). Do organic inputs alter resistance and resilience of soil microbial community to drying?. Soil Biology and Biochemistry.

[ref-46] Oliveira MT, Medeiros CD, Frosi G, dos Santos MG (2014). Different mechanisms drive the performance of native and invasive woody species in response to leaf phosphorus supply during periods of drought stress and recovery. Plant Physiology and Biochemistry.

[ref-47] Pan L, Zhang X, Wang J, Ma X, Zhou M, Huang L, Nie G, Wang P, Yang Z, Li J (2016). Transcriptional profiles of drought-related genes in modulating metabolic processes and antioxidant defenses in *Lolium multiflorum*. Frontiers in Plant Science.

[ref-48] Pan Y, Wu LJ, Yu ZL (2006). Effect of salt and drought stress on antioxidant enzymes activities and SOD isoenzymes of liquorice (Glycyrrhiza uralensis Fisch). Journal of Plant Growth Regulation.

[ref-49] Parida AK, Dagaonkar VS, Phalak MS, Umalkar GV, Aurangabadkar LP (2007). Alterations in photosynthetic pigments, protein and osmotic components in cotton genotypes subjected to short-term drought stress followed by recovery. Plant Biotechnology Reports.

[ref-50] Puranik S, Jha S, Srivastava PS, Sreenivasulu N, Prasad M (2011). Comparative transcriptome analysis of contrasting foxtail millet cultivars in response to short-term salinity stress. Journal of Plant Physiology.

[ref-51] Purcell L, Andyking C (1996). Drought and nitrogen source effects on nitrogen nutrition, seed growth, and yield in soybean. Journal of Plant Nutrition.

[ref-52] Qin C, Yu C, Shen Y, Fang X, Chen L, Min J, Cheng J, Zhao S (2014). Whole-genome sequencing of cultivated and wild peppers provides insights into Capsicum domestication and specialization. Proceedings of the National Academy of Sciences of the United States of America.

[ref-53] Quan R, Shang M, Zhang H, Zhao Y, Zhang J (2004). Improved chilling tolerance by transformation with betA gene for the enhancement of glycinebetaine synthesis in maize. Plant Science.

[ref-54] Ramachandra RA, Chaitanya KV, Vivekanandan M (2004). Drought-induced responses of photosynthesis and antioxidant metabolism in higher plants. Journal of Plant Physiology.

[ref-55] Ramanjulu S, Bartels D (2002). Drought- and desiccation-induced modulation of gene expression in plants. Plant, Cell and Environment.

[ref-56] Sahitya UL, Krishna MSR, Deepthi RS, Prasad GS, Kasim DP (2018). Seed antioxidants interplay with drought stress tolerance indices in Chilli (*Capsicum annuum* L) seedlings. BioMed Research International.

[ref-57] Sánchez-González M, ICRD V, GM G (2007). Generalized height-diameter and crown diameter prediction models for cork oak forests in Spain. Forest Syestems.

[ref-58] Saxon EC, Groves CR (2003). Adapting ecoregional plans to anticipate the impact of climate change. Drafting a conservation blueprint: a practitioner’s guide to planning biodiversity.

[ref-59] Scherr SJ, Mcneely JA (2008). Biodiversity conservation and agricultural sustainability: towards a new paradigm of ‘ecoagriculture’ landscapes. Philosophical transactions of the Royal Society of London. Series B, Biological Sciences.

[ref-60] Serraj R, Sinclair TR, Purcell LC (1999). Symbiotic N_2_ fixation response to drought. Journal of Experimental Botany.

[ref-61] Shubhra Dayal J, Goswami CL, Munjal R (2004). Influence of phosphorus application on water relations, biochemical parameters and gum content in cluster bean under water deficit. Biologia Plantarum.

[ref-62] Sicher RC, Timlin D, Bailey B (2012). Responses of growth and primary metabolism of water-stressed barley roots to rehydration. Journal of Plant Physiology.

[ref-63] Smith MD (2011). The ecological role of climate extremes: current understanding and future prospects. Journal of Ecology.

[ref-64] Spehn EM, Scherer-Lorenzen M, Schmid B, Hector A, Caldeira MC, Dimitrakopoulos PG, Finn JA, Jumpponen A, Ódonnavan G, Pereira JS, Schulze ED, Troumbis AY, Körner C (2002). The role of legumes as a component of biodiversity in a cross-European study of grassland biomass nitrogen. Oikos.

[ref-65] Sun F, Pan K, Olatunji OA, Li Z, Chen W, Zhang A, Song D, Sun X, Huang D, Tan X (2019). Specific legumes allay drought effects on soil microbial food web activities of the focal species in agroecosystem. Plant and Soil.

[ref-66] Sun F, Pan K, Tariq A, Zhang L, Sun X, Li Z, Wang S, Xiong Q, Song D, Olatunji OA (2016). The response of the soil microbial food web to extreme rainfall under different plant systems. Scientific Reports.

[ref-67] Temperton VM, Mwangi PN, Scherer-Lorenzen M, Schmid B, Buchmann N (2007). Positive interactions between nitrogen-fixing legumes and four different neighbouring species in a biodiversity experiment. Oecologia.

[ref-68] Tezara W, Mitchell VJ, Driscoll SD, Lawlor DW (1999). Water stress inhibits plant photosynthesis by decreasing coupling factor and ATP. Nature.

[ref-69] Tong PY (1994). Achievements and perspectives of tillage and cropping systems in China. Cropping System and Cultivation Technology.

[ref-70] Veselin P, Jacques H, R BM, Gechev TS (2015). ROS-mediated abiotic stress-induced programmed cell death in plants. Frontiers in Plant Science.

[ref-71] Wang JY (2016). Coupling effects of water and fertilizer on diurnal variation of photosysnthesis of *Zanthoxylum bungeanum Maxim* ‘hanyuan’ seedling leaf. Scientia Horticulturae.

[ref-72] Wang Z, Silva LCR, Sun G, Luo P, Mou C, Horwath WR (2015). Quantifying the impact of drought on soil-plant interactions: a seasonal analysis of biotic and abiotic controls of carbon and nutrient dynamics in high-altitudinal grasslands. Plant and Soil.

[ref-73] Xia L, Zhao F, Mao K, Yuan Z, Zuo Z, Xu T (2018). SPI-based analyses of drought changes over the past 60 years in China’s major crop-growing areas. Remote Sensing.

[ref-74] Xin-Ping Chen Z-LC, Vitousek PM, Cassman KG, Matson PA, Bai J-S, Meng Q-F, Hou P, Yue S-C, RÖmheld V, Zhang F-S (2011). Integrated soil-crop system management for foof security. Proceedings of the National Academy of Sciences of the United States of America.

[ref-75] Yang F, Feng L, Liu Q, Wu X, Fan Y, Raza MA, Cheng Y, Chen J, Wang X, Yong T, Liu W, Liu J, Du J, Shu K, Yang W (2018). Effect of interactions between light intensity and red-to- far-red ratio on the photosynthesis of soybean leaves under shade condition. Environmental and Experimental Botany.

[ref-76] Yingchao L, Yin X, Xiao J, Tang L, Zheng Y (2019). Interactive influences of intercropping by nitrogen on flavonoid exudation and noculation in faba bean. Scientific Reports.

[ref-77] Yuan H, Wang H, Wang L, Chai L, Tian C (2017). Nutritional evaluation and functional properties of the antioxidant polypeptide from Zanthoxylum bungeanum Maxim seeds kernel protein hydrolysate. CYTA—Journal of Food.

[ref-78] Zhou Y, Lam HM, Zhang J (2007). Inhibition of photosynthesis and energy dissipation induced by water and high light stresses in rice. Journal of Experimental Botany.

[ref-79] Zhou YH, Yu JQ, Huang LF, Nogués S (2004). The relationship between CO_2_ assimilation, photosynthetic electron transport and water–water cycle in chill-exposed cucumber leaves under low light and subsequent recovery. Plant, Cell and Environment.

